# Overcoming the pitfalls of categorizing continuous variables in ecology, evolution and behaviour

**DOI:** 10.1098/rspb.2024.1640

**Published:** 2024-10-02

**Authors:** Roxanne S. Beltran, Corey E. Tarwater

**Affiliations:** ^1^ Department of Ecology and Evolutionary Biology, University of California, 130 McAllister Way, Santa Cruz, CA 95060, USA; ^2^ Department of Zoology and Physiology, University of Wyoming, 1000 East University Avenue, Laramie, WY 82071, USA

**Keywords:** statistics, breakpoint, threshold, category, bin, quantitative ecology

## Abstract

Many variables in biological research—from body size to life-history timing to environmental characteristics—are measured continuously (e.g. body mass in kilograms) but analysed as categories (e.g. large versus small), which can lower statistical power and change interpretation. We conducted a mini-review of 72 recent publications in six popular ecology, evolution and behaviour journals to quantify the prevalence of categorization. We then summarized commonly categorized metrics and simulated a dataset to demonstrate the drawbacks of categorization using common variables and realistic examples. We show that categorizing continuous variables is common (31% of publications reviewed). We also underscore that predictor variables can and should be collected and analysed continuously. Finally, we provide recommendations on how to keep variables continuous throughout the entire scientific process. Together, these pieces comprise an actionable guide to increasing statistical power and facilitating large synthesis studies by simply leaving continuous variables alone. Overcoming the pitfalls of categorizing continuous variables will allow ecologists, ethologists and evolutionary biologists to continue making trustworthy conclusions about natural processes, along with predictions about their responses to climate change and other environmental contexts.

## 1. Introduction

Variation in morphology, physiology and behaviour is ubiquitous among organisms [1]. Moreover, these traits interact with environmental and ecological factors to drive individual differences in behaviour, survival and reproductive success [2]. Many studies are designed to characterize these intraspecific patterns and their mechanistic drivers; however, the interpretation of data depends critically on how variables are measured and analysed [3,4]. Imagine you are a graduate student measuring lizards to determine whether larger lizards are more likely to inhabit shadier patches. You spend countless hours catching and placing lizards against a measuring tape… 43 mm, 46 mm, 42 mm, 41 mm, 48 mm… 500 lizards later, you have a normal distribution with an impressive sample size. You decide to categorize lizards into ‘small’, ‘medium’ and ‘large’ because boxplots are visually appealing and commonly used in the literature. The 42 mm lizard is placed in the ‘medium’ category and the 41 mm lizard is placed in the ‘small’ category. Are the two lizards really *that* different in size? What if your measurement error is ±3 mm because measuring active lizards in the hot desert sun is challenging? Would you feel comfortable recommending a land manager focus on small lizards (<42 mm) because only that size group did not use shady habitat in the same way as the other groups? What if we told you that by keeping your lizard size measurements as continuous predictors, all those long fieldwork days would pay off with sufficient statistical power and much stronger interpretation?

The lizard example highlights a prevalent issue in the fields of ecology, evolution and behaviour—categorizing variables that are continuous during data collection or analysis. Pitfalls of categorizing continuous variables (regardless of whether the continuous variables are linear or nonlinear) have been well-documented in the statistics and biomedical literature, including loss of information about differences between individuals, the assumption that all individuals within a given group respond the same way to the variable of interest, reduced power of statistical tests resulting in the need for a larger sample size to detect differences, lack of ability to make comparisons or conduct meta-analyses in studies that categorize variables in different ways, risk of overlooking nonlinear effects, increased probability of type I and II errors and poor predictive ability [5–9]. Perhaps most importantly, conclusions made from inappropriately categorized variables may not only be misleading, but may also be wrong [7,10,11]. Despite well-recognized pitfalls of categorization, the practice persists in published research. Many researchers still categorize continuous predictor variables under the assumption that this is a better practice for small sample sizes, skewed data, potentially distinct groups and measurement intensive variables, or because they believe the simplicity of interpretation outweighs the costs of categorization [5]. Scientists encourage mentees and students to develop *a priori* predictions for their research that often involve categorizing continuous predictor variables for simplicity. However, analysing variables as continuous does not preclude testing of these categorical predictions. Further, while management decisions, much like biomedical ones, are often dichotomies (to treat/not treat a symptom, to list/not list a species as endangered), this does not mean predictor variables should be analysed as dichotomies.

Our goal is to create a guide on improving data collection, analysis and interpretation that we wish we had when we first tackled statistical analyses in independent research projects. To do this, we: (i) review the prevalence and current practices of categorizing continuous variables; (ii) discuss when or if categorization is appropriate using specific examples of common predictor variables; (iii) provide three examples from simulated field data to show how statistical power, model fits and interpretation are impacted by categorization of a continuous variable using various categorization breakpoints (e.g. median, arbitrary intervals), nonlinear data and different sample sizes; and (iv) provide a step-by-step guide on how to keep variables continuous throughout the entire scientific process. The manuscript and its associated code could be used as a practical laboratory for students and teachers to develop valuable programming and research skills including data simulation, plotting, model comparisons and interpretation.

## 2. Current practices in categorizing continuous variables

### (a) Mini-review methods

To examine the frequency with which researchers categorize continuous predictor variables, we evaluated 72 publications in six popular journals: *Proceedings of the National Academy of Sciences* (Ecology and Evolution section), *Ecology*, *Evolution*, *Journal of Experimental Biology*, *Behavioral Ecology* and *Animal Behaviour*. We chose these journals because they were a mix of high-impact general and subject-specific journals, and included both observational and experimental studies. Starting with the newest issue available, we examined the 12 most recent empirical articles in which continuous predictor variables were included in the final model (electronic supplementary material, table S1). Twelve articles were chosen to obtain a sufficient sample size per journal and across all journals. Publications (*n* = 98) were excluded from the review (i.e. not included in the tally) if they did not have a predictor variable in the final model that could be treated as continuous, or if they were theoretical, meta-analyses, or phylogenetic analyses. The final set of publications varied in taxa (e.g. birds, arthropods, reptiles, mammals, plants) and response variables (e.g. molting, occupancy, leaf damage, niche breadth, fecundity), and included both experiments and observational studies.

### (b) Frequency of categorization

**Figure 1.**
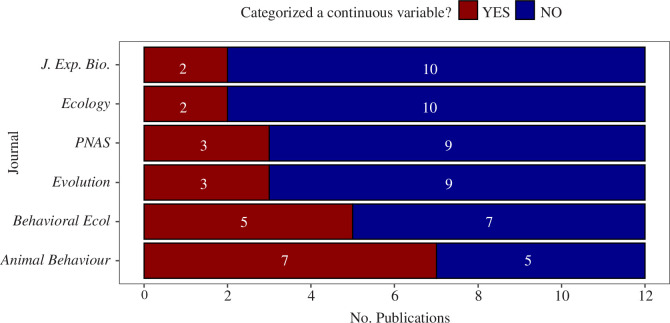
The number of publications in a selection of science journals that include categorized continuous variables, ordered by prevalence. *J. Exp. Biol., Journal of Experimental Biology; PNAS, Proceedings of the National Acadamy of Sciences; Behavioral Ecol., Behavioral Ecology*.

Of the 72 articles, 31% included categorical predictor variables in their final model that could have been left as continuous data. Although we acknowledge that in behaviour, researchers often use ordinal scales that group different behaviours, and these may be kept as categories, we did not observe these types of scales as predictor variables in our review. Instead, the presence of categories was most often related to habitat and distance. The frequency of categorization varied substantially among journals and was notably higher in the behaviour journals as compared to the other journals (figure 1). Nevertheless, sample sizes in our mini-review were small; a larger review would be needed to adequately determine which discipline has more prevalent categorization. Here, our take-away is that categorizing predictor variables is a common practice across disciplines.

Our estimate of categorization was an underestimate of the true categorization present in journal publications because we took a narrow sense of categorization. We only counted an article as a ‘yes’ for categorizing a continuous predictor variable if that predictor variable was included in the final model. Nevertheless, in many of the articles, categorization came much earlier on in the data processing stage or during data collection (and thus the publication was assigned as ‘no’ for categorization in our review or was not included if continuous predictor variables were absent in the final model). For example, one study used a 10 cm cut-off to categorize whether a conspecific was ‘near’ versus ‘far’ during data collection and near/far were used as a categorical predictor variable [12]. This article was not assigned a ‘yes’ for categorization because categorization was done during the data collection phase.

### (c) What we learned

Across the 22 publications that categorized continuous variables, the justification for choosing a breakpoint (where scientists decide to split the data into categories) varied widely from citing previous studies as precedent, biological rationale, statistical rationale, arbitrary rationale or no rationale at all. Statistical reasons were the most common and were based on a variety of breakpoints, including: intervals (flexible number of categories through even increments across range of values), median (two categories split by 50% point in data), uneven (three categories,<25%, 25–75%, >75%), quartiles (four categories, 0–25%, 26–50%, 51–75%, 76–100%) and bimodal (two categories, ‘natural’ break in portion of distribution with no values). Biological reasons varied, but tended to be based on elevation, habitat, diet and season [13–18]. Justification for choosing a particular breakpoint was rarely provided, even in the case of statistical rationale (e.g. why did the researcher choose quartiles instead of another method?).

Although we did not include experiments with categorical treatments as a ‘yes’ in terms of being a study that categorized continuous predictor variables, experimental treatments were often based on categorizing continuous variables (e.g. high or low temperature treatments) and many researchers made decisions to categorize continuous predictor variables in the early stages of data processing. For example, re-using the study mentioned above, the authors set a breakpoint to define whether a conspecific animal was near (<10 cm) or far (>10 cm) from the focal individual and then used this value to create a forced categorical predictor variable in the study, rather than using distance itself [12]. If 10 cm is based on the sensory cues of the species (e.g. perhaps an individual cannot detect individuals farther than 10 cm away), then a breakpoint may be justified, but in this case, a justification was not given. One experimental study showed the original trait distribution of hindlimb length in lizards and then justified their groupings (short versus long hindlimb length) by choosing the extremes of the trait distribution [19]. However, for most experiments, justifications of treatments were not given. Justification of breakpoints is critically important to facilitate transparency and ensure reproducibility, which are critical to the future of our field [20,21].

## 3. Should continuous variables ever be categorized?

### (a) Cases where categorization may be justified

There is rarely a good reason to categorize continuous predictor variables, irrespective of what statistical method is used [7,9]. However, there are some cases in which there exists solid justification or no other choice, depending upon the research question. For example, studies frequently use existing trait databases, such as Mammal Diversity Database, FishBase and AVONET, that often categorize traits and species (e.g. migratory strategy, diet, dominant habitat). In these situations, obtaining continuous data is difficult (although see EltonTraits [22] for an example of a database that estimates foraging attributes continuously). Land cover classes (e.g. grassland, woodland, bare soil), land use classes (e.g. agricultural land, urban), ocean zones (e.g. epipelagic, mesopelagic, bathypelagic) and ecoregions are other examples of commonly categorized data. These categories may be justified, depending upon the research question and study design constraints. However, in many cases, continuous data are better suited to address research questions and even if using these categories, distance to a particular land or water class may make more biological sense given we know that edge effects (changes that occur at the boundary of two or more habitats) are fundamentally important [23]. For example, categorizing the habitats of two animals into ‘forest’ when one is on the forest/grassland boundary and the other is in the middle of the forest may result in misleading conclusions about the impact of habitat on fitness (for an example, see Powell *et al.* [24,25]). Likewise, using distances to particular land use and cover classes may be informative for understanding at what point animals are impacted by these habitats [23]. Thus, many of the studies that use land or water classes often use both the categories alone (e.g. likelihood a bird species is found in riparian habitat) and a continuous variable of distance (e.g. likelihood a bird species is a given distance from riparian habitat) because these two variables may tell a different story. For example, Ribic *et al*. [26] relate seabird species’ densities, biomass and diversity to categorical water masses (High Antarctic versus Low Antarctic water mass), but augment the categorical data with a continuous analysis of distance to respective water masses.

Categorization may be justified if the research question is focused on a mechanistic break, such as whether an animal is foraging on/off the continental shelf or above/below tree line. Still, we urge researchers to think critically about the rationale behind these breakpoints. For example, the distance from shore to the continental shelf varies globally, and while abiotic variables, species behaviour and predation rates may change rapidly near the shelf, edge effects still exist. Similarly, when thinking about modelling (above/below) tree line, we encourage researchers to think about the precise question of interest. Is the researcher simply interested in tree line, such that they want to group together all variables that are associated with tree line, or does the researcher care about disentangling the mechanisms (e.g. changes in temperature or moisture levels during the growing season [27])? Further, similar to the continental shelf, tree lines can occur at various elevations and can be abrupt boundaries or transition zones [27]. These different factors and the precise research question need to be considered when deciding whether to categorize predictors, even when mechanistic breaks are used.

### (b) How commonly categorized predictor variables can be examined continuously

Table 1 describes predictor variables that are often categorized, provides examples of how they could be analysed as continuous, and cites an example paper that analysed each as continuous.

**Table 1.** Common covariates to bin, and examples of publications (including response variable, predictor variable, study species, citation) that avoid pitfalls of categorizing continuous variables. (Note that these publications are a broader set than those included in our mini-review.)

predictor variable	categories	continuous	example of continuous
**abiotic**			
temperature	cold versus warm	degree Celsius per unit time	molt timing~water temperature, lobsters [28]
climate state	El Niño versus neutral versus La Niña	index	body growth~climate indices, sea turtles [29]
rainfall	dry versus wet	millimetres rainfall per unit time	activity levels~daily precipitation, ungulates [30]
season	summer versus winter, dry versus wet	day of year	foraging behaviour~day of year, manta rays [31]
**anatomy/morphology**			
body size	large versus small	centimetres	abundance~body size, marine and terrestrial vertebrates and invertebrates [32]
**physiology/phenology**			
diving performance	shallow versus deep	metres depth	lifetime fitness~dive depth, seals [33]
life-history timing	early versus late	days since life-history event	diving depth~weeks since fledging, penguin [34]
**behaviour**			
personality	bold versus shy	exploration score	faecal cortisol~exploration score, squirrels [35]
diet	frugivore versus granivore	% of diet	fledging rate~diet (% occurrence of garbage), birds [36]
migration/dispersal distance	near versus far	metres	body mass loss~dispersal distance, beetles [37]
landscape habitat	high versus low	metres high	height growth~elevation, trees [38]
seascape habitat selection	coastal versus offshore	kilometres from coast	abundance~distance from coast, whales [39]
aeroscape habitat selection	canopy versus midstory versus understory	altitude (metres above the ground)	predation risk~altitude, birds [40]
site fidelity	low versus high	Bhattacharyya’s affinity	variability in mass gain~site fidelity index, seals [41]
diurnal activity patterns	day versus night	solar position	diving depth~solar illumination, fur seals [42]
**demography**			
age	young versus old	years	behaviour and physiology~age, birds [43]
quality	high versus low	lifetime reproductive success (LRS)	aging~LRS, birds [44]
lifespan	short versus long	years	LRS~reproductive lifespan, birds [45]

### (c) Additional details on commonly categorized predictors

Based on our mini-review, we wish to provide additional detail about how two specific predictors (a third on diet is in electronic supplementary materials) were categorized, and underscore why the biological rationale provided may not be appropriate.

#### (i) Elevation

It was common to bin elevation (high/low), rather than using the continuous variable of metres above sea level. However, it is well-known that abiotic and biotic variables change along elevational gradients [46]. If there are only two study sites, one higher and one lower in elevation, then using elevation is synonymous with using study site as a categorical predictor. However, if multiple study sites exist, we suggest including elevation as a continuous predictor, or include other variables (e.g. slope, aspect, oxygen and temperature) to get closer to the mechanisms driving patterns. Even if study sites are grouped with a few at higher elevations and a few at lower elevations, as long as there is some variation between them, using elevation or continuous abiotic variables is often preferred over categorization. In addition to providing more statistical power, changes in the variables of interest may not be the same within similar elevation sites because we often do not know the scale at which abiotic and biotic variables are changing. For example, in a bird species, there are substantial differences in the demography of two nearby populations owing to only a 1–2 m difference in elevation [47–49]. In this habitat, a 1 m change is the difference between being seasonally flooded versus dry [49]. Likewise, variables may change in inconsistent and nonlinear ways, with threshold effects potentially occurring at different elevations [50,51]. In these situations, testing for nonlinearity in the continuous predictor variables using generalized additive models (GAMs) or fitting different polynomial terms (e.g. quadratic, cubic) would allow the evaluation of how environmental variables change with elevation, without making assumptions about how to categorize the environmental variables (and whether those variables are linear). Although not discussed here, other environmental metrics such as aspect should also remain continuous when possible.

#### (ii) Season

Seasons are often categorized based on abiotic conditions or life cycle events. For example, seasons may be based on abiotic conditions, such as dry/wet, or they may be based on breeding/non-breeding or growing/non-growing. Seasons based on abiotic conditions are challenging because the definition of the start and end of a season is often arbitrary and the end of a particular season may be more similar to the start of the next season than to its own season [52]. Instead, we suggest using day of year rather than season to reduce bias [52]. If day of year is not a suitable variable owing to how data were collected, then month could suffice and still be analysed continuously. If the main research question is focused on seasonal changes, then we suggest picking the mechanistic variable that is thought to be driving patterns, and using that data to define a meaningful variable of season. For example, Rutt & Stouffer [53] were interested in changes in interaction networks across seasons and they followed Li & Fu [54] and Fu *et al*. [55] to empirically determine the onset of the wet season based on hourly precipitation data. We urge researchers to keep in mind that when using day of year and other similar variables, these are cyclic in nature (day 365 is closely related to day 1) and should be analysed as such (e.g. GAMs with a cyclic cubic regression spline). When seasons are based on life stages, we would argue that ideally, they would be based on individuals being followed rather than what is typically the beginning and end of seasons. For example, many studies show the start and end dates of seasons are highly variable [47,56], and with climate change, fluctuations in the timing of seasons are predicted to worsen [57,58].

## 4. Examples

### (a) Background

We simulated data to quantify the detrimental impact of categorizing continuous variables using various statistical breakpoints and sample sizes (details below). To give the example biological relevance, we created a dataset that illustrates the complexity of life-history theory and climate change impacts, and contains a predictor variable that is frequently categorized (table 1)—reproductive timing in one year and its effect on body mass in the following year. This simple model has only one predictor. A reasonable research question would be: how does timing of reproduction in year *t* influence body mass at the start of the breeding season in year *t* + 1? For illustrative purposes, let us say we collected data from individually banded penguins in Antarctica. Based on the mechanistic relationships between seasonally available sea ice and food availability, we hypothesize that late reproductive timing could negatively impact the abilities of penguins to accumulate body mass before the next breeding season. Let us say we wander around the penguin colony recording the initiation date of first nest of each banded penguin (reproductive timing, measured as ‘day of year’, continuous), and then return to Antarctica the following year to weigh those same penguins using a platform scale (body mass, kilograms, continuous). For all simulations, we used a Gamma distribution from the rgamma() function, which randomly generates numbers with a Gamma distribution from specified shape and scale parameters, because our data are positive continuous and we would expect a long distributional tail where there are only a few tiny breeding penguins. We conducted two linear simulations shown below and a nonlinear simulation (electronic supplementary materials). Further details on modeling can be found in the electronic supplementary materials. With these data, we will answer the questions: how does the relationship between reproductive timing and body mass change if reproductive timing data are categorized using different breakpoints, and the dataset contains different sample sizes? We used R v. 4.3.1 [59] for all analyses. For the linear models, we used the glm() function (Gamma, link=′log′) for model fitting, the AIC() function for Akaike information criterion model comparisons [60], the emmeans() function to calculate 95% confidence intervals (CIs ±1.96 * s.e. back-transformed from the log scale), and the cld() function to create compact letter displays. We calculated *R* ^2^ using McFaddens’ pseudo-*R* ^2^ [61]. Code to reproduce data, analyses and figures is available at [62,63].

### (b) Simulated categorization breakpoints in a linear continuous variable

#### (i) Model performance

**Figure 2.**
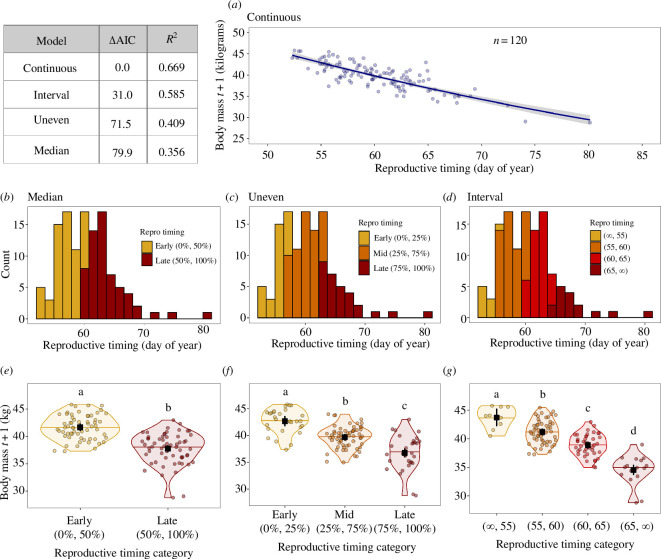
Forced categorization of a continuous predictor variable (reproductive timing) leads to poorer model fits (using AIC), wider 95% CIs and lower variance explained (*R* ^2^) compared with a continuous model. In the continuous plot, the predicted line and CI ribbon are shown. In the categorical plots (median, uneven, interval), coloured violin plots show the kernel probability densities (outlines) and medians (horizontal lines) of the data at different categories, coloured circles show (jittered) body mass values within each reproductive timing category and black squares show predicted output of each model with error bars (black lines) representing the upper and lower 95% CIs. Two or more predictor levels with the same grouping letter were not different at the *α* = 0.05 level.

We categorized the continuous predictor variable reproductive timing that we ‘collected’ (figure 2*a*) with three breakpoints commonly found in publications: median (figure 2*b,e*), uneven (figure 2*c,f* ) and interval (figure 2*d,g*). The continuous model outperformed all methods of categorization as indicated by lower AIC and higher proportion of variance explained (*R* ^2^) (figure 2; table 1). The CIs around the continuous predictor was relatively low (1.29 at the mean predictor) and the CIs around the categorical predictors became progressively larger with more categories, as the number of estimated parameters increased. For example, the range in CIs (upper CI – lower CI) for the earliest reproductive timing category in each method spanned 1.39 in the median example, 1.95 in the uneven example and 3.09 in the interval example (figure 2*e*–*g*). Depending on the breakpoint method used, a single value (e.g. 60th day of the year) could be assigned to several different categories (e.g. ‘late’ using the median method, ‘mid’ using the uneven method and ‘60–65’ using the interval method). Likewise, categorizing continuous variables often increases the number of estimated parameters, which is penalized by AIC and functionally reduces the sample size. Note: we include multiple commonly used model comparison methods (AIC, *p*-values) for illustrating the results, but do not recommend using them together. Likewise, we include *R* ^2^, but do not recommend using *R* ^2^ to decide the ‘best’ model. In figure 2*f*,*g*, the small letters above the plots indicate the *p*-values based on pairwise comparisons.

#### (ii) Model interpretation

In this penguin example, the continuous predictor variable would be used to conclude that individuals that begin breeding later in the year have smaller body masses the following year (slope or *β* in model = 0.985, meaning that body mass decreases by 1.5% every day their breeding start is delayed), suggesting that there is a cost to breeding later. Specifically, a 10 day later start to reproduction (e.g. a shift from a reproductive timing of day 55 (the 10th percentile) to day 65 (the 90th percentile)) would be associated with a 14% smaller body mass the following year (from 43 to 37 kg; figure 2*a*). We would also conclude that 67% of the variance in body mass could be explained by reproductive timing the previous year. By contrast, the categorical predictor methods would be used to conclude that 36–59%, depending upon method, of the variance in body mass could be explained by reproductive timing the previous year. For the median categorization model, we would conclude that individuals which start breeding between days 60 and 81 have lower body mass (mean mass: 38 kg) the following year compared with individuals that start breeding days less than 60 (mean mass: 41 kg). For the uneven categorization model, we would conclude that individuals that start breeding between days 63 and 80 (mean mass: 37 kg) have lower body bass the following year compared with individuals that start breeding earlier (days 57–63, mean mass: 40 kg), and compared with individuals that breed even earlier (days: <57, mean mass: 43 kg). For the interval categorization model, the general interpretation is the same, but what keeps changing in each of these categorization models is the day of year in which individuals have lower body mass, and what that mean body mass is.

#### (iii) Conclusions

In this scenario, irrespective of the statistical method used, penguins that breed later in the year have lower body mass the following year than individuals that breed earlier in the year. However, what is lost in the interpretation when using categorical predictors is that the reduction in body mass declines consistently with timing of reproduction. Thus, individuals within each of the categories are not experiencing the same cost of reproduction. Further, understanding how costs of reproduction (in terms of reduced body mass) change with reproductive timing is unclear with categorization. Does an individual that starts breeding at day 60 pay a much greater cost than an individual that starts breeding at day 61? Or, does an individual that starts breeding at day 63 pay the same cost as an individual that waits until the very end of the breeding season (day 80)?

### (c) The sample size argument

#### (i) Model performance

**Figure 3.**
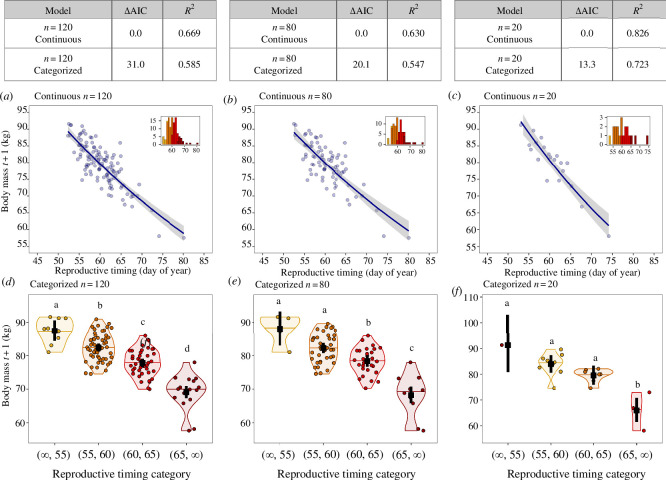
Forced categorization of a continuous predictor variable leads to poorer model fits (using AIC), higher *p-*values, and larger 95% CIs compared with a continuous model, especially for datasets with small sample sizes. Inset histograms show the distributions of data in each category, with the same *x-*axis limits and ticks as figure 3*a*–*c*. The meanings of colours, shapes and lines are given in the figure 2 legend.

We repeated the linear simulation/prediction for three different sample sizes: *n* = 120 (as in §4b), and subsetted from that original dataset to samples of *n* = 80 and *n* = 20, ensuring the range of reproductive timing data were similar across sample size subsets. We compare performance of only two predictor variables: continuous and interval (because it was the most common method in our review). Across sample sizes, the continuous model outperformed the interval model in terms of model fit (figure 3; table 1).

#### (ii) Model interpretation

In this penguin example, the continuous predictor variable would be used to conclude that the later an individual breeds in the year, the lower their body mass the following year (figure 3*a*). Further, reproductive timing strongly influenced body mass the following year, with 63–69% of the variance in body mass being explained by reproductive timing, depending on the sample size. By contrast, with the categorical methods, 55–63% of the variance in body mass was explained by reproductive timing, depending on sample size. With the smaller sample size (*n* = 20), the categorical variable (categorized method) would be used to conclude that there was no significant relationship between reproductive timing and body mass in year *t* + 1 for penguins that bred before day 65. Similarly, with the middle sample size (*n* = 80), the interpretation would be that a reduction in body mass the following year occurred for individuals that start breeding after day 60, but no differences were found for those that started breeding earlier in the year.

#### (iii) Conclusions

While small sample sizes are often used as a reason to categorize continuous variables, it is clear from this example that this justification is not valid. The conclusions drawn from the categorized models would be fundamentally different from those drawn from the continuous models for smaller sample sizes. In particular, only individuals breeding late in the year would appear to pay a cost of reproduction in terms of reduced body mass the following year, while those breeding early in the year are all the same and do not pay costs. When treating reproductive timing as continuous, the interpretation does not change with sample size. This is not true when treating reproductive timing as categorical; the interpretation changes with each change in sample size.

## 5. A step-by-step guide

### (a) Research question development

The most common challenge we see when working with early career researchers is developing precise, testable research questions so that the analysis can match the research question (this is not as easy as it sounds!). We suggest that researchers sketch out theoretical graphs to solidify the variables, questions, relationships and analyses. Do they predict threshold effects or other types of nonlinear relationships, or interactions between variables? Do they want to make predictions about climate change impacts? For example, if they want to know what may happen to sea urchin growth rates with each degree change in temperature, they should analyse the continuous relationship between the two. Everything we discuss below needs to be thought of in light of the specific research question.

### (b) Study design

When designing the study, the researcher should recall that many variables exist along a continuum, rather than in categories and that appropriate methods should be applied [64]. This can help determine the number and location of plots, treatments, study duration and data collection methods. For example, if the research question centres on environmental gradients, having more than two field sites is critical. During our review, we discovered that researchers publishing in the journal *Ecology* often did a terrific job of collecting data from at least three sites along a particular environmental gradient [65]. The general rule of thumb is one needs at least 8–10 rows of data for each predictor variable in a model to obtain a sufficient sample size [66], depending upon the spread in the data and other factors. However, keep in mind that categorizing variables reduces power and requires larger sample sizes [67]. For example, dichotomizing a continuous predictor is roughly equivalent to losing a third of the data, making it more difficult to detect relationships [7]. Further, if a researcher is designing an experiment, we would argue that (depending on the researcher’s goals) experiments do not need to have categorical treatments. If categorical treatments are going to be used, they should be clearly and carefully justified.

### (c) Data collection

If data can be collected in a continuous way in the field or laboratory, we strongly suggest doing so. We acknowledge that sometimes researchers may collect data in bins owing to the challenges of data collection. For example, when counting fruit on a tree, researchers often count in bins (e.g. 0–10, 10–20, 20–30, 30–40, 40–50 fruits) because it is challenging and time-consuming to count each individual fruit. Nevertheless, even if one must categorize owing to feasibility issues, the data do not need to be analysed this way. For example, if one can only count fruit in groups of 10 (as in the above example), in the analysis stage, these groupings can still be analysed as continuous (e.g. 5, 15, 25, 35, 45) rather than categorical. Biologically, this often makes more sense. For example, frugivores are often attracted to trees with more fruit, and thus we would expect that the greater the amount of fruit, the more visitations by frugivores [68]. We do not expect that the bin of 0–10 fruits is going to similarly impact visitation as the bin of 40–50 fruits; instead, we expect a continuous increase in visitation with the number of fruits (up to a certain point).

### (d) Data processing and analyses

Aside from experiments with *a priori* categorical treatments, data processing and analyses were the two stages in which categorization of continuous predictors was observed most often in our mini-review. In general, we rarely recommend categorizing predictors. However, if it is going to be done it needs to be well justified and based on a clear theoretical, mechanistic or biological reason. We recommend never choosing a categorization breakpoint to minimize the *p*-value or AIC value or maximize the *R* ^2^. The examples we showed above were simple, and based on having only one predictor that had a linear or nonlinear relationship with the response variable. In reality, most of our research includes multiple predictors, and statisticians have shown that the more predictors there are in a model, the worse it is to categorize [7]. Here, we show a straightforward GAM to illustrate that the same challenges of categorizing continuous data arise irrespective of whether the relationships are linear or nonlinear (electronic supplementary material). Researchers often do not test for nonlinearity, but we argue that often relationships are not linear. In this case, researchers should analyse data using techniques that appropriately deal with nonlinear functional forms, such as GAMs, as we have done here, or fitting polynomial terms. In these cases, we recommend being aware of sample size limitations, and constraining the number of knots in a GAM to avoid overfitting the data. Categorizing continuous variables often results in the loss of these nonlinear relationships, particularly when using methods with larger categories (e.g. median or uneven method). Alternatively, some researchers may categorize continuous predictors precisely because they detect nonlinear relationships. Nevertheless, this presents its own challenges, such as using additional degrees of freedom if adding more categories, and deciding *a priori* where the shifts in the slope occur, rather than allowing the data to speak for themselves (as occurs with GAMs). We suggest that researchers look at whether there are nonlinear relationships prior to development of the final model including model selection.

### (e) Reporting/manuscript drafting

Irrespective of research question development, study design, data collection and data processing/analysis, the most critical step is to be explicit about what was done and why (see Santoro *et al*. [69] for a good example). In our mini-review, there were few cases where we could determine why a particular breakpoint was selected and in some cases, we could not determine if a predictor was analysed as continuous or categorical. There were some cases where the researcher said that the decision of a particular breakpoint was arbitrary. While we appreciate honesty, categorization needs to be well-justified given that it can drastically change interpretations. There is almost no statistical rationale for categorizing continuous data [5–9]. Therefore, other reasons need to be made clear. We also appreciate that many journals have strict word limits that make it difficult to detail all analytical steps in the main manuscript; appendices or electronic supplementary material may be used instead.

## 6. Discussion

### (a) Summary

Here, we summarized the prevalence and practice of categorizing continuous variables in ecology, behaviour and evolutionary biology and provide guidelines for overcoming those pitfalls. Specifically, we showed that categorizing continuous variables is common (30% of literature reviewed in six popular journals; figure 1), and that many frequently used predictor variables—including abiotic, morphological, physiological, behavioural and demographic—can be collected and analysed continuously (table 1). We also demonstrated how categorizing continuous variables can lower statistical power and change interpretation using a simulation of field data, and that smaller sample sizes only make it worse (not better as is commonly believed; figures 2 and 3). Further, we showed that while the rationale for using a particular breakpoint (e.g. quartile versus median) is rarely given, the choice of a breakpoint may alter interpretation (figures 2 and 3). Finally, we provided recommendations on how to keep variables continuous throughout the entire scientific process (§5). Together, these insights provide an actionable guide to avoiding misleading scientific conclusions. The resulting continuous analyses can be used to provide valuable insights, including into individual differences in traits and responses, increased statistical power, smaller required sample sizes and facilitation of large synthesis studies [5–9]. We hope our step-by-step guide and code will be useful to both teachers and graduate students alike. Below, we emphasize a few key points about categorizing predictors and the scientific process.

We acknowledge that researchers face challenging predicaments when compromising between statistical perfection and feasibility given limited time, resources and data. In this manuscript, we did not attempt to assign severity to instances of categorization (e.g. distinguish negligence from justifiable situations). Across the wide range of study designs, sample sizes and statistical tests in ecology, behaviour and evolutionary biology, categorization has different impacts from threatening inference to only minimal changes in inference. Nevertheless, we strongly encourage researchers to avoid categorizing continuous variables when feasible, unless justification is solid, owing to the many pitfalls associated with this practice.

### (b) You can still talk about groups even when analysing continuous predictors

When developing *a priori* predictions or presenting results (either for a conference or in a manuscript), categorizing to simplify and improve interpretation is common, and valid. Even if a predictor is analysed as continuous, a researcher can still discuss the extremes (e.g. earlier versus later reproductive timing, see our examples). For example, suppose our *a priori* prediction was that late breeding penguins would pay a higher cost of reproduction while early breeders would pay little to no cost (§4b). We do not need to categorize our data into early and late to test this prediction. Instead, we discussed the change in body mass across reproductive timing, highlighting that breeding later in the year is more costly in terms of reduced body mass in year *t* + 1 compared with breeding earlier. We also made direct comparisons between those breeding earlier (10th percentile) and those breeding later (90th percentile) in terms of impacts on body mass in year *t* + 1. Similarly, Maitland & Rahel [70] show a good example of how trophic diversity varies along a longitudinal stream gradient (analysed continuously), but they graphically demonstrate and discuss in their results the different habitats of headwaters, foothills and great plains. Thus, researchers can still make comparisons of different points in the continuous graph based on extremes or percentiles (e.g. using quantile regression [71]) to highlight that various points along the range are responding differently. Similarly, if two continuous predictors are interacting in the final model, it is valid for ease of interpretation to show a graph of one predictor as continuous across the *x*-axis and categories (e.g. high, medium and low) of the second predictor variable, even though the second predictor variable is analysed as continuous [72]. The argument that continuous predictors make interpretation difficult or do not allow one to test predictions is not a solid justification.

### (c) The world is neither linear nor abrupt all the time

Understanding the shapes of relationships is critically important for correctly interpreting results and for making management decisions. Here we showed a simple nonlinear relationship (Supplemental Materials), where basic interpretation between the nonlinear continuous method versus categorical methods was similar. Nevertheless, if we had a relationship that was more nonlinear (which may be expected, for example, when time is a predictor), differences in interpretation would be even more dramatic. Categorizing continuous predictors can obscure nonlinear relationships, while at the same time leading to the assumption that abrupt transitions exist between variables. Nevertheless, gradual transitions and nonlinear relationships are common in nature. Studying transitions in environmental and ecological gradients can provide insight into the differences among individuals, populations, and communities, and the mechanisms underlying these differences. Thus, there is much knowledge to be gained by not assuming linearity nor abrupt transitions, and we would argue for the need to test for both when going through the analysis stage of the scientific process.

### (d) Summary and conclusions

The human brain is hard-wired to place items into categories [73]. However, much of the research in ecology, behaviour and evolutionary biology over the past few decades has clearly demonstrated that individual variation on a continuous scale is ubiquitous and important [74]. As a result, it is often problematic to assume that individuals within a group have the same traits, are doing the same thing or will respond in the same way [75]. Likewise, work across environmental gradients has been incredibly informative, but there is still much we do not understand in terms of the shapes of relationships and their underlying mechanisms [76]. Falsely categorizing continuous predictors hampers our ability to make future predictions and to address questions of conservation importance. We encourage researchers to think critically about their research questions prior to starting analyses (and even better, prior to collecting data!). If a categorization decision cannot be justified (e.g. with a mechanistic break, only data available), then it should not be done. Additionally, we strongly recommend researchers look for nonlinear relationships, as these often occur in nature. We are not asking researchers to go against their temptation to categorize—categorization during interpretation can be fine—but instead to understand that many variables exist along continuums and must be analysed continuously. Keeping predictor variables continuous lets the data speak for themselves, while still allowing the flexibility to interpret results or make predictions.

## Data Availability

Code to reproduce data, analyses, and figures is available at [62, 63]. Supplementary material is available online [77].
